# Primary malignant peripheral nerve sheath tumor of the liver: a case report and literature review

**DOI:** 10.3389/fonc.2026.1795231

**Published:** 2026-06-12

**Authors:** Xue Hu, Lei Zhou, Yue Tian, XingLan Li, Tao Lu

**Affiliations:** 1 Department of Radiology, Sichuan Provincial People’s Hospital, University of Electronic Science and Technology of China, Chengdu, China; 2 Department of Radiology, People's Hospital of Shizhu Tujia Autonomous County, Chongqing, China; 3 Department of Pathology, Sichuan Provincial People’s Hospital, University of Electronic Science and Technology of China, Chengdu, China

**Keywords:** case report, computed tomography, liver, malignant peripheral nerve sheath tumor, primary hepatic tumor

## Abstract

**Background:**

Malignant peripheral nerve sheath tumor (MPNST) is a malignant neoplasm originating from Schwann cells or other nerve sheath components. It most commonly arises in the extremities and trunk. Primary hepatic MPNST is extremely rare, and most cases are associated with neurofibromatosis type 1 (NF-1).

**Case presentation:**

This study reports a rare case of hepatic MPNST without NF-1 in a 56-year-old female. The patient presented with upper abdominal pain and distention. Serum tumor markers were all within normal ranges. Radiological examination identified a hypovascular hepatic lesion, which was pathologically confirmed as MPNST. Radical tumor resection was performed. The patient has been followed up for approximately 2 years, with no signs of recurrence or metastasis.

**Conclusion:**

The clinical manifestation of hepatic MPNST is nonspecific with normal tumor markers. Radiologically, it mainly manifests as a hypovascular tumor, which requires differentiation from hepatocellular carcinoma, intrahepatic cholangiocarcinoma, and hepatic angiosarcoma. With a literature review, our article provides a comprehensive summary of its CEUS and CECT manifestations, thereby supplementing the limited available data on this rare entity.

## Introduction

Malignant peripheral nerve sheath tumor (MPNST) is a malignant neoplasm derived from Schwann cells or other nerve sheath cells and accounts for 5%-10% of soft tissue sarcomas, with neurofibromatosis type 1 (NF-1, von Recklinghausen’s disease) accounting for roughly 50% of cases (3, 12). The most commonly involved sites of the disease include the nerve roots and nerve tracts in the extremities and trunk, especially the sciatic nerve (3, 6–9). Visceral involvement by this tumor is rare, with reported cases occurring in the heart, lungs, cervix, ovaries, and other organs (13–20). Primary hepatic MPNST is extremely rare.

A literature search was conducted in PubMed and Web of Science up to January 2026. The literature search strategy was as follows: (“malignant peripheral nerve sheath tumor” OR “MPNST” OR “malignant schwannoma”) AND “liver”. The inclusion criteria were as follows: (1) Confirmed primary hepatic MPNST cases with pathological diagnosis; (2) Cases with complete clinical and/or imaging data; (3) Original articles published in English. To our knowledge, only 11 cases of primary hepatic MPNST have been described in the English literature, among which 9 were not associated with NF-1. However, none of these previous reports provided detailed descriptions of the imaging features of this tumor. In this study, we describe another rare case of primary hepatic MPNST without NF-1 in a 56-year-old female, including comprehensive imaging findings, with a review of previous literature.

## Case presentation

A 56-year-old female was admitted to our hospital with upper abdominal pain and distention for 20 days. She had no history of cirrhosis, jaundice, viral hepatitis, or other malignancies, but had a history of hypertension for 6 years, treated with amlodipine besylate and enalapril maleate. The patient had lost approximately 5 kg of weight since disease onset. No abnormal findings were noted on physical examination. Neurological and dermatological examinations were normal. Serum tumor markers, including alpha-fetoprotein (AFP), carcinoembryonic antigen (CEA), and carbohydrate antigen 19-9 (CA19-9), were all within normal ranges.

Ultrasonography demonstrated a slightly hyperechoic, irregular mass in the left hepatic lobe, measuring 7.6 cm × 7.1 cm with a well-defined margin and internal vascularity on pre-contrast imaging. In contrast-enhanced phases, the mass exhibited slightly earlier enhancement in both the periphery and center compared to the surrounding hepatic parenchyma in the arterial phase. At peak enhancement, the lesion demonstrated mildly hyperenhanced echogenicity with moderate homogeneity. The mass had heterogeneous iso-enhancement in the portal venous phase and mild washout in the delayed phase (approximately 3 minutes) (**Figure 1**).

**Figure 1:**
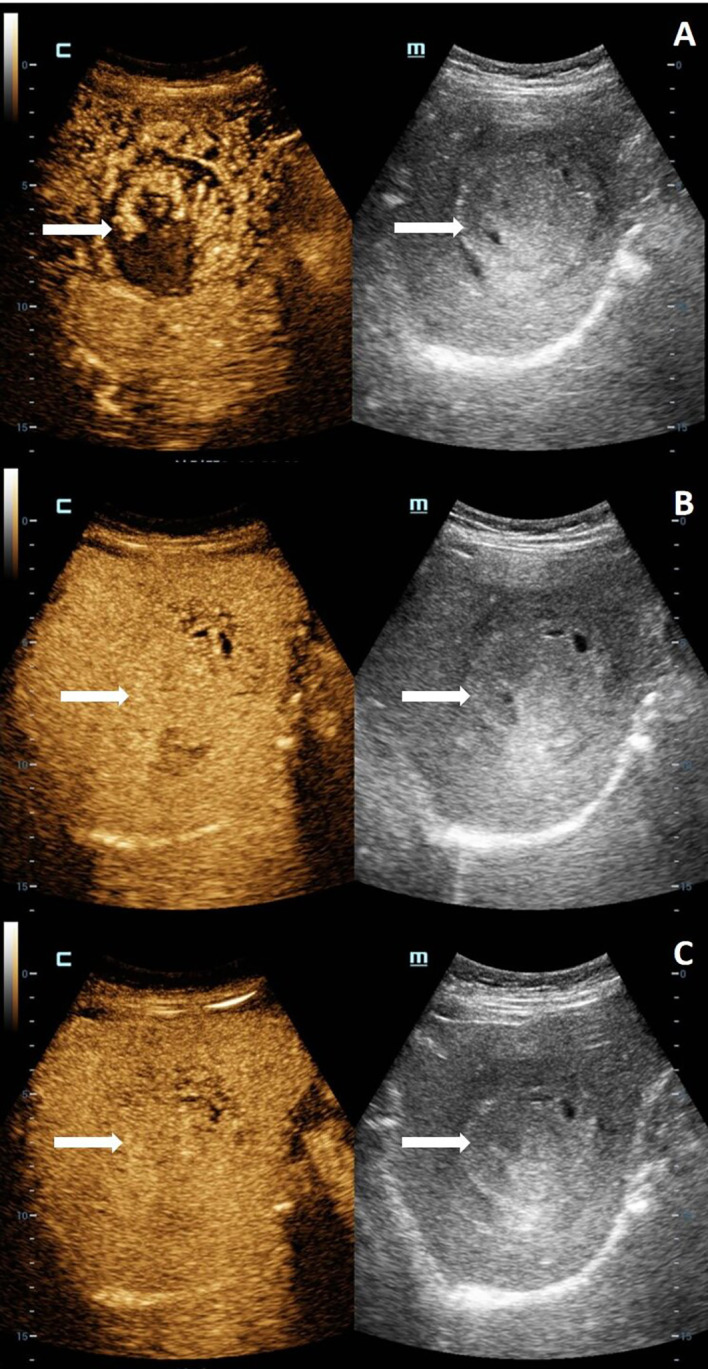
CEUS images. **(A)** Arterial phase showed a mass exhibited slightly earlier enhancement at both the periphery and center compared to the surrounding hepatic parenchyma. **(B)** Portal venous phase showed the mass had heterogeneous iso-enhancement. **(C)** Delayed phase showed the mass had mild washout.

Contrast-enhanced CT (CECT) revealed a well-defined, heterogeneous mass in the left hepatic lobe, measuring approximately 7.0 cm × 6.1 cm. The mass was hypoenhanced in the arterial phase, with curved tumoral arteries in the center. In the portal venous and delayed phases, the mass showed persistent, mild heterogeneous enhancement, with no invasion of the hepatic veins or portal vein. Neither intrahepatic nor extrahepatic biliary dilatation or obstruction was identified (**Figure 2**). Three−dimensional (3D) reconstruction of abdominal CT images showed that the mass was supplied by the left hepatic artery, with compression of the left and middle hepatic veins (**Figure 3**).

**Figure 2:**
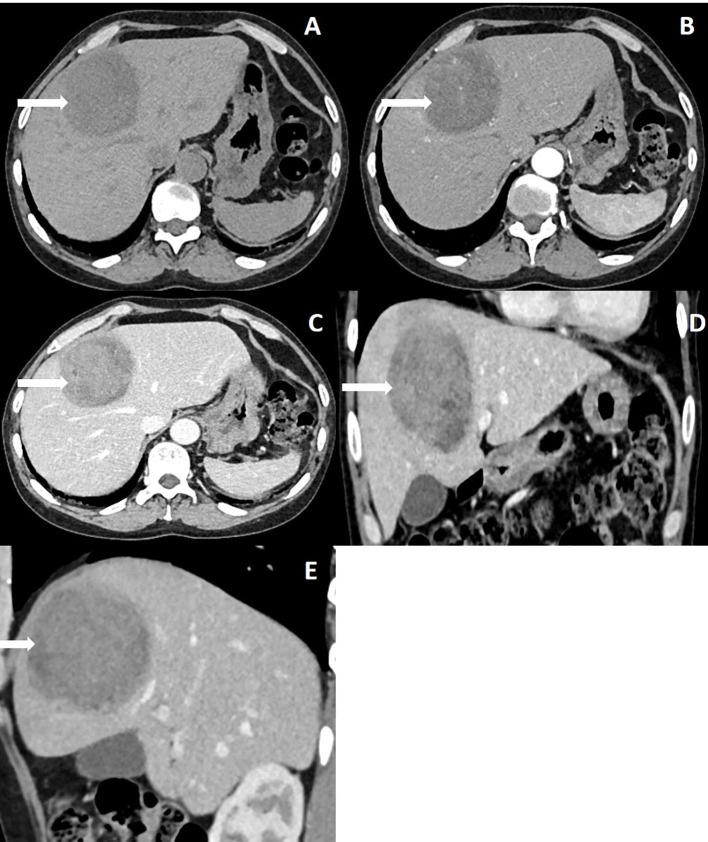
CT images. **(A)** Non-enhanced CT image showed a well-defined and heterogeneous mass in the left lobe of the liver. **(B)** Axial arterial phase image showed the mass was hypoenhanced. **(C)** Axial portal phase image showed the mass had persistent and heterogeneous slight enhancement with no invasion of the hepatic veins or portal veins. **(D, E)** Coronal and sagittal delayed phase images showed the mass had persistent slight enhancement.

**Figure 3:**
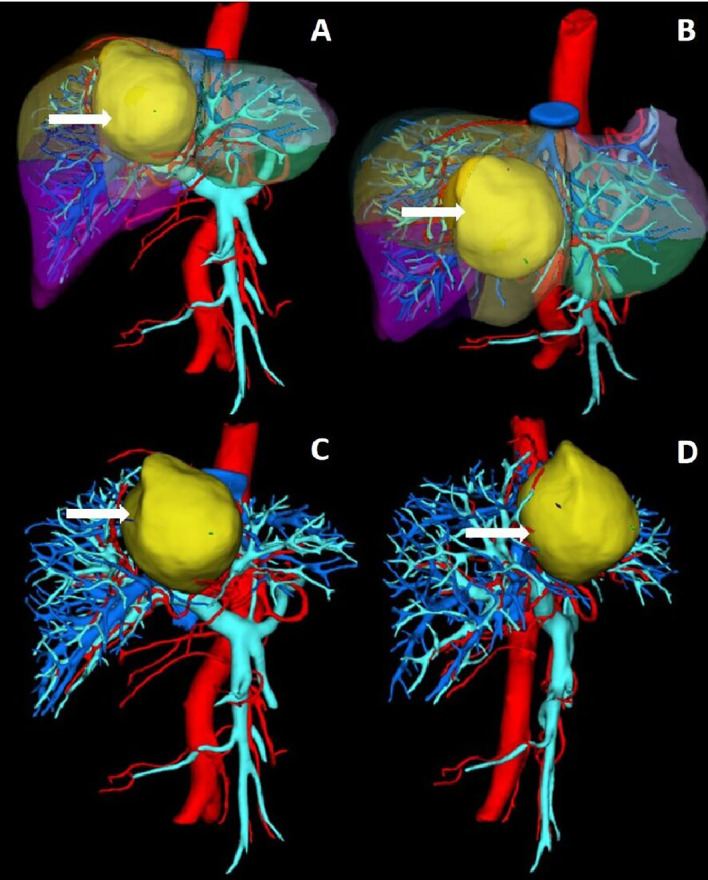
3D reconstruction of CT images of the liver. The tumor was labeled in yellow (indicated by the white arrow), the abdominal aorta and hepatic artery were labeled with red, the portal vein and its branches were labeled with cyan, and the hepatic vein and inferior vena cava were labeled with blue.

A left hemihepatectomy was performed on this patient. During surgery, a soft tumor measuring approximately 6.0 cm × 5.0 cm was identified in segments 3 and 4 of the liver. The tumor had an ill−defined margin and flesh-like internal tissue. No obvious metastatic lesions were detected in the abdominal cavity, peritoneum, or omentum. Hematoxylin-eosin (H&E) staining showed that the tumor was predominantly composed of spindle-shaped cells, which were irregular in shape with twisted, wavy, comma-shaped, or tadpole-like nuclei. Prominent nucleoli were present, and the cytoplasm was eosinophilic. Mitotic figures were frequently observed. Hypercellular areas alternated with hypocellular areas, forming a “marble-like” or whorled pattern. Tumor cells frequently aggregated around blood vessels, suggesting perivascular growth. Immunohistochemical staining was performed for further characterization. Tumor cells were positive for S-100 and SOX-10, with a Ki-67 labeling index of 30% (**Figure 4**), but negative for CD117, CD34, CK, DOG1, DES, EMA, SMA, STAT6, TLE1, pTRK, caldesmon, Melan-A, HMB-45, myogenin, ER, Cam5.2, p63, ALK-1, CK19, CK7, and ERG. Based on these findings, the pathological diagnosis of MPNST was confirmed. The patient has undergone regular postoperative CT follow-up, and no obvious signs of recurrence or metastasis have been identified to date. The complete clinical timeline for this patient is shown in **Figure 5**.

**Figure 4:**
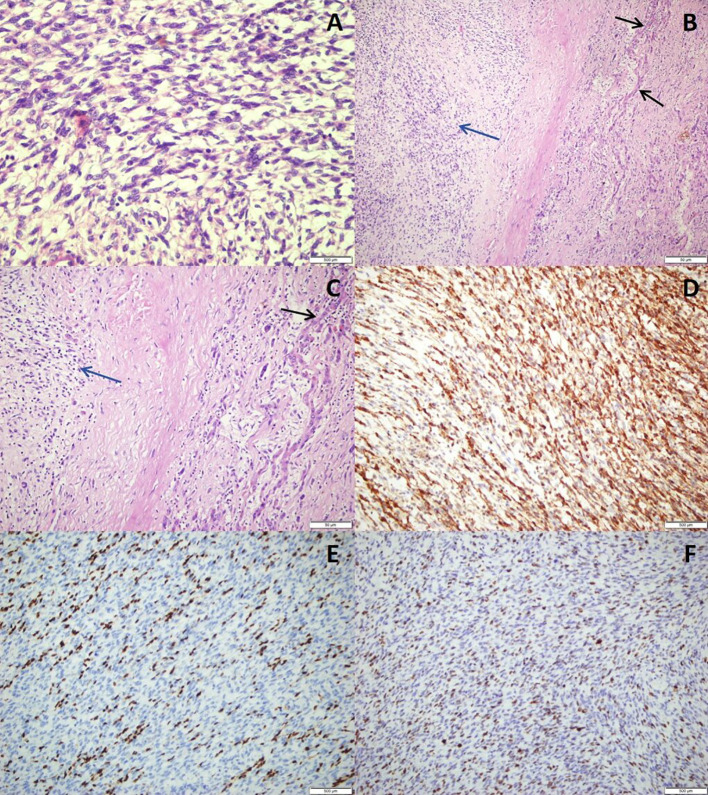
**(A)** H&E revealed the tumor was composed predominantly of spindle-shaped cells. Prominent nucleoli were present; the cytoplasm was eosinophilic. Some areas showed increased numbers of blood vessels. Original magnification: 40×. **(B, C)** H&E revealed both normal hepatic tissue and tumor tissue simultaneously. The blue arrow indicates the tumor, and the black arrow marks a small amount of residual liver tissue. **(B)** Original magnification: 10×; **(C)** Original magnification: 20×. **(D)** The tumor cells were positive for S-100. Original magnification: 20×. **(E)** The tumor cells were positive for SOX-10. Original magnification: 20×. **(F)** Immunohistochemistry showing 30% positivity for Ki-67. Original magnification: 20×.

**Figure 5:**
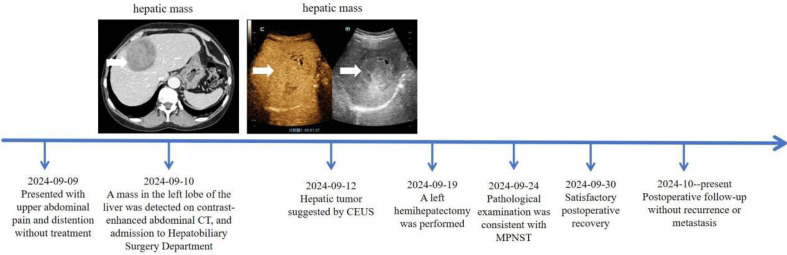
Timeline of the patient.

## Discussion

MPNST is one of the most common malignant mesenchymal tumors of the soft tissues (3). It accounts for approximately 5–10% of all soft tissue sarcomas and arises in association with peripheral nerves, neurofibromas, and schwannomas, often in association with NF-1 (7, 8, 21, 22). The most common anatomical sites for MPNST are the upper and lower extremities and trunk, whereas visceral involvement is exceedingly rare (8). Previously reported visceral manifestations of this tumor have primarily involved the heart, lungs, cervix, and ovaries, while primary hepatic involvement is extremely rare (13–20). Occurrence in the liver in patients without NF-1 is even rarer. To our knowledge, only 9 cases of primary hepatic MPNST not associated with NF-1 have been reported in the English literature (**Table 1**) (1–11). In this study, we describe another rare case of primary hepatic MPNST without NF-1 in a 56-year-old female.

**Table 1:** Clinical characteristics of patients with MPNST of the liver.

Reference	Age/sex	Symptoms	Neurofibromatosis type 1 (NF-1)	Size (cm)	Immunohistochemistry	Treatment	Outcome
Young SJ 1975 (1)	23/M	Right hypochondrial pain	Yes	20	N/A	N/A	died of disease
Lederman SM 1987 (2)	21/M	Right upper quadrant pain and swelling	Yes	30	S-100(-)	N/A	died of disease
Morikawa Y 1995 (3)	63/M	Severe abdominal pain	No	20 × 13	S-100 (+), Vimentin (+)	received no treatment	died of disease
Fiel MI 1996 (5)	49/M	Jaundice	No	15	S-100 (+), Vimentin (+)	radical tumor resection	alive
Sheikh MY 1996 (4)	35/F	Abdominal distension, weight loss, and abdominal pain	No	N/A	N/A	N/A	N/A
Iddings DM 2008 (6)	83/M	No symptoms	No	7	S-100 (+)	radical tumor resection	alive
Kóbori L 2008 (7)	22/F	Right upper quadrant pain and extreme edema	No	26	S-100 (+), Vimentin (+)	extended right hemihepatectomy	alive
Subramaniam K 2012 (8)	71/M	Right upper quadrant pain and abdominal distension, fever and night sweats	No	21	S-100 (+), Vimentin (+)	received one cycle of palliative chemotherapy and subsequently declined further treatment	N/A
Kakizaki S 2016 (9)	72/F	Enlarging epigastric mass	No	18	S-100 (+), Vimentin (+)	conservative treatment	died of disease
Jung HI 2016 (11)	33/F	Right flank pain	No	12.5 × 11	S-100 (+), Vimentin (+)	right hepatectomy	alive
Benjamin J 2022	20/F	Shortness of breath and back pain	No	11	S-100 (+), SOX-10 (+)	N/A	N/A
Present case	56/F	Upper abdominal pain and swelling, weight loss	No	7 × 6.1	S-100 (+), SOX-10 (+)	left hemihepatectomy	alive

F, Female.

M, Male.

N/A, Not Available.

The median age of onset was 42 years (range, 20–83 years), with a male-to-female ratio of 1:1. The most common clinical symptom was upper abdominal pain, followed by abdominal distention and jaundice. These manifestations are nonspecific and are similar to those of other common hepatic malignancies. Only one previously reported case was asymptomatic. Tumor markers are also nonspecific for diagnosis. AFP and CEA are typically normal, while CA19–9 may be slightly elevated in some patients with biliary obstruction. In the present case, a middle-aged female presented with abdominal pain and normal tumor marker levels, which is similar to previous cases.

Histologically, MPNST is typically characterized by spindle cells arranged in fascicles with irregular nuclear contours (3–9). Mitotic figures are frequently identified, and the cytoplasm typically displays eosinophilic staining (3, 6–10). Densely cellular areas alternate with prominent stromal regions, and loose myxoid foci may also be present (6–8). However, in the majority of cases, these features are often insufficient to confirm the diagnosis. Further confirmation of its neural origin relies on immunohistochemical examination. S-100 protein is positive in 50%–90% of MPNSTs but lacks complete specificity, as it may also be expressed in other spindle cell neoplasms, including synovial sarcoma, leiomyosarcoma, and rhabdomyosarcoma (23). SOX-10 typically exhibits focal or patchy positivity in MPNSTs, although expression may be absent in high-grade or poorly differentiated tumors. The histopathological and immunohistochemical features in the current case were consistent with classic MPNST. The primary histological differential diagnoses include primary hepatic leiomyosarcoma and fibrosarcoma. Leiomyosarcoma consists of fascicular spindle cells and expresses SMA, desmin, and vimentin but is negative for S-100 (24). Fibrosarcoma shows a classic herringbone pattern of spindle cells and is positive for vimentin but negative for desmin, actin, Syn, and SMA (25).

Among the reported cases, hepatic MPNST occurs more frequently in the right hepatic lobe (7 of 9 cases) (1–3, 6, 8, 9, 11). Most lesions present as masses with a diameter greater than 5 cm, and some tumors exceed 20 cm. Our case was in the left hepatic lobe with a diameter of more than 7 cm, representing an uncommon hepatic location. Only three previous cases have described the grayscale ultrasonic features of this tumor, which mainly manifested as heterogeneous hypoechoic masses, occasionally containing mixed hyperechoic or cystic components (**Table 2**) (2, 3, 9). However, none of the previous reports described the contrast-enhanced ultrasound (CEUS) manifestations, making the present case the first to document its perfusion pattern. The ultrasound contrast agent consists of gas microbubbles, which are distributed exclusively within capillaries and the blood pool without extravasation into the interstitial tissue. This permits real-time visualization of blood perfusion and microcirculation of MPNST during the arterial phase, whereas fibrous stroma and necrotic areas show no enhancement (26, 27). Compared with CT, CEUS provides dynamic, real-time evaluation of the overall perfusion characteristics of hepatic lesions (28). In the present case, the tumor exhibited slightly early enhancement in the arterial phase, heterogeneous iso-enhancement in the portal venous phase, and mild washout in the delayed phase (approximately 3 minutes). This perfusion pattern directly reflects the tumor’s underlying microvascular architecture and stromal composition.

**Table 2:** Imaging features of patients with hepatic MPNST.

Reference	Location	Vascular compression	Ultrasonography	Precontrast CT	Arterial phase	Venous phase	Delayed phase
Young SJ 1975 (1)	both lobes	N/A	N/A	N/A	N/A	N/A	N/A
Lederman SM 1987 (2)	right lobe	N/A	inhomogeneous but predominantly hypoechoic	N/A	N/A	N/A	N/A
Morikawa Y 1995 (3)	right lobe	common hepatic artery and right hepatic artery	inhomogeneous but predominantly hypoechoic	inhomogeneous hypodense	not enhanced	N/A	N/A
Fiel MI 1996 (5)	central liver	the main portal vein	N/A	N/A	N/A	N/A	N/A
Sheikh MY 1996 (4)	N/A	N/A	N/A	N/A	N/A	N/A	N/A
Iddings DM 2008 (6)	segments 4, 5 and 8	portal vein	N/A	low density	N/A	N/A	N/A
Kóbori L 2008 (7)	segment I–III	N/A	N/A	inhomogeneous	N/A	N/A	N/A
Subramaniam K 2012 (8)	right lobe	N/A	N/A	complex solid and cystic	N/A	N/A	N/A
Kakizaki S 2016 (9)	entire lobe	N/A	combined hyperechoic and cystic pattern	solid with several low density areas	not enhanced	N/A	N/A
Jung HI 2016 (11)	right lobe	N/A	N/A	hypodense mass and contained a punctuate calcification	heterogenous enhanced	N/A	N/A
Benjamin J 2022 (EMPNST)	N/A	N/A	N/A	N/A	N/A	N/A	N/A
Present case	left lobe	None	slightly hyperechoic	heterogeneous	hypoenhanced	heterogeneous slight enhancement	persistent slight enhancement

CT, Computed Tomography.

N/A, Not Available.

On CT, hepatic MPNST typically appears as a heterogeneous hypodense mass (**Table 2**) (3, 7–10), with rare cystic changes or calcifications. Two previous studies showed no significant tumor enhancement after contrast administration (3, 9). However, the present case demonstrated predominantly hypovascular features with persistent mild enhancement until the delayed phase, and without invasion of the hepatic veins, portal vein, or bile ducts, representing an imaging pattern distinct from those in previous reports. Pathologically, these imaging features are attributable to abundant collagen fibers in the tumor stroma and low microvascular density, allowing the CT contrast agent to slowly diffuse from vascular endothelium into the tumor interstitium, thereby producing persistent mild enhancement. CECT facilitates evaluation of tumor vascularity, which is critical for assessing tumor biological aggressiveness and guiding clinical treatment strategies (29).

Hepatic MPNST is extremely rare and requires differentiation from intrahepatic cholangiocarcinoma (CCA), hepatocellular carcinoma (HCC), and hepatic angiosarcoma (HAS). (1) Intrahepatic cholangiocarcinoma (iCCA): iCCA arises from biliary epithelium and usually demonstrates a mass-forming growth pattern (30, 31). It is frequently associated with elevated serum CA19–9 levels (32). On CT, iCCA typically appears as a large non-capsulated hypodense mass with peripheral duct dilation and peripheral enhancement in both arterial and venous phases with late centripetal spread (30, 33). However, the mass in our case lacked peripheral biliary dilatation and showed no progressive centripetal enhancement, which is distinctly different from the typical imaging features of iCCA.

(2) Hepatocellular carcinoma (HCC): HCC is the most frequent primary malignancy of the liver, strongly associated with chronic hepatitis B virus infection and liver cirrhosis (34). Serum AFP is frequently elevated in affected patients (34, 35). On CECT, most HCCs show the hallmark “arterial hyperenhancement with venous washout” pattern: marked homogeneous or heterogeneous enhancement in the arterial phase, followed by hypoattenuation relative to the surrounding hepatic parenchyma in the portal venous and delayed phases (35). In contrast, the present case showed arterial hypoenhancement with persistent mild enhancement in the portal venous and delayed phases, which is inconsistent with the typical enhancement pattern of HCC. Moreover, the patient had no history of chronic hepatitis B virus infection or cirrhosis, and the AFP level was within the normal reference range, further distinguishing this case from conventional HCC.

(3) Hepatic angiosarcoma (HAS): Primary HAS is an extremely rare malignancy, accounting for only 4% of all angiosarcomas regardless of origin and less than 1% of all primary hepatic malignancies (36–38). No specific serum markers are routinely used for diagnosis. On non-contrast CT, HAS typically shows as a heterogeneous hypodense mass, often containing hyperdense foci from hemorrhage or necrosis (39, 40). On CECT, HAS classically demonstrates peripheral rim enhancement in the arterial phase, with progressive centripetal fill-in during the delayed phase (36, 39). These imaging features are distinctly different from the mild arterial enhancement and persistent mild heterogeneous enhancement in the portal venous and delayed phases observed in the present case.

Radical resection with wide negative margins remains the primary treatment for MPNST (41, 42). Patients with incomplete excision of the tumor may show a significantly higher risk of local and distant recurrence than those undergoing complete tumor resection (43). MPNST is relatively resistant to radiotherapy and chemotherapy. There is currently insufficient evidence to confirm that these treatments can enhance the prognosis for patients with MPNST (44). Studies have suggested that radiotherapy may effectively control local disease and reduce the rate of local recurrence in MPNST (45). However, its impact on improving overall survival benefits remains a subject of debate (45, 46).

The prognosis for patients with MPNST is extremely poor, with a 5-year survival rate ranging from 16% to 52% (47). Among MPNST patients undergoing surgical resection, those with negative surgical margins had a 5-year survival rate of 67%, compared to 22% for those with positive margins (48). Important adverse prognostic factors for MPNST include tumor size greater than 10 cm, stage III or higher, high tumor grade with more than 20 mitoses per 10 high-power fields, association with NF-1, mesenchymal pathological subtype, prior radiotherapy, and positive surgical margins (46). Among the 11 previously reported cases of primary hepatic MPNST, the ratio of survival to death was 1:1 (with 3 cases lacking follow-up data) (**Table 1**). The present case was followed up for approximately 2 years with no signs of recurrence observed.

Hepatic MPNST presents notable diagnostic challenges owing to its rarity and non-specific clinical and radiological features. Clinically, patients often have only vague abdominal symptoms, normal serum tumor markers, and no history of chronic liver disease. Radiologically, non−specific hypovascular enhancement patterns on CEUS and contrast−enhanced CT mimic other hepatic malignancies. Definitive diagnosis depends on postoperative histopathological and immunohistochemical examinations. Diffuse S−100 and SOX−10 positivity confirms nerve−sheath origin, while negative staining for other markers excludes alternative spindle-cell neoplasms.

The main strengths of this report are as follows: First, although US is a commonly used modality for hepatobiliary disease clinically, our case is the first to describe the CEUS features of primary hepatic MPNST, which supplies a preliminary evaluation of the tumor. Second, the CT manifestations of the tumor were poorly described in previous literature. Our case showed the tumor was mainly solid with mild enhancement. Based on a comprehensive evaluation of the tumor using both CEUS and CECT, although being rare, MPNST should be included in the diagnosis for patients presenting with upper abdominal pain and distention, no history of chronic hepatitis or cirrhosis, and normal serum AFP levels.

Despite the above strengths, this study also has certain limitations. First, the study adopts a single-case design, which is inherently limited by the lack of a large sample size, making it difficult to generalize the findings to all patients with primary hepatic MPNST. Second, the generalizability of the results is limited. Due to the rarity of the disease, we cannot exclude the possibility of individual differences in the clinical manifestations, imaging findings and disease progression of different patients. Third, the number of previously reported cases of primary hepatic MPNST is small, and the literature data are limited, which may affect the comprehensive analysis of the disease’s clinical characteristics and prognosis.

In conclusion, we report a rare case of primary hepatic MPNST without NF-1 in a 56-year-old female and describe its clinical, radiological, and pathological features, thereby supplementing the limited literature. Based on the small number of previously published cases, the prognosis of primary hepatic MPNST appears dismal, with most reported cases demonstrating a highly aggressive clinical course characterized by recurrence, metastasis, and mortality (1–11). Its non-specific clinical and imaging features often lead to misdiagnosis, thus emphasizing the value of detailed case reports for improved clinical recognition and differential diagnosis.

## Data Availability

The raw data supporting the conclusions of this article will be made available by the authors, without undue reservation.
