# Correction: Primary malignant peripheral nerve sheath tumor of the liver: a case report and literature review

**DOI:** 10.3389/fonc.2026.1923986

**Published:** 2026-07-13

**Authors:** Xue Hu, Lei Zhou, Yue Tian, XingLan Li, Tao Lu

**Affiliations:** 1 Department of Radiology, Sichuan Provincial People’s Hospital, University of Electronic Science and Technology of China, Chengdu, China; 2 Department of Radiology, People’s Hospital of Shizhu Tujia Autonomous County, Chongqing, China; 3 Department of Pathology, Sichuan Provincial People’s Hospital, University of Electronic Science and Technology of China, Chengdu, China

**Keywords:** case report, computed tomography, liver, malignant peripheral nerve sheath tumor, primary hepatic tumor

Author Lei Zhou was erroneously omitted as equal contributing author. The original version of this article has been updated.

